# Bedside ultrasound education in Canadian medical schools: A national survey

**Published:** 2016-03-31

**Authors:** Peter Steinmetz, Octavian Dobrescu, Sharon Oleskevich, John Lewis

**Affiliations:** 1 Department of Family Medicine, McGill University, Montreal, QC; 2 Faculty of Medicine, McGill University, Montreal, QC

## Abstract

**Background:**

This study was carried out to determine the extent and characteristics of bedside ultrasound teaching in medical schools across Canada.

**Methods:**

A cross-sectional, survey-based study was used to assess undergraduate bedside ultrasound education in the 17 accredited medical schools in Canada. The survey, consisting of 19 questions was pilot-tested, web-based, and completed over a period of seven months in 2014.

**Results:**

Approximately half of the 13 responding medical schools had integrated bedside ultrasound teaching into their undergraduate curriculum. The most common trends in undergraduate ultrasound teaching related to duration (1–5 hours/year in 50% of schools), format (practical and theoretical in 67% of schools), and logistics (1:4 instructor to student ratio in 67% of schools). The majority of responding vice-deans indicated that bedside ultrasound education should be integrated into the medical school curriculum (77%), and cited a lack of ultrasound machines and infrastructure as barriers to integration.

**Conclusions:**

This study documents the current characteristics of undergraduate ultrasound education in Canada.

## Introduction

Bedside ultrasound (point-of-care ultrasound) is being integrated into clinical practice as an adjunct to the physical exam and patient history. As ultrasound becomes an essential element of the clinician’s bedside assessment, it is being introduced into the undergraduate medical school curriculum.^1^,^2^

The benefits of an undergraduate ultrasound education are evident in studies in which students show better diagnostic accuracy and estimation of organ size when using bedside ultrasound in combination with physical examination, as compared with specialists using physical examination alone.^3^,^4^ Physical examination skills are enhanced by the use of ultrasound in 88% of second-year students, while 100% of first- to fourth-year students agreed that the ultrasound teaching they received would help them in future specialties.^6^ Undergraduate ultrasound education is most commonly implemented to help students better understand anatomy 1,7–18 as evidenced by first-year medical students with ultrasound teaching performing significantly better than ultrasound naïve students on an anatomy test,^19^ and by 84% of first-year medical students stating that ultrasound teaching improved their understanding of three-dimensional anatomy.^20^ In spite of the described benefits, some believe that teaching bedside ultrasound is not appropriate at the undergraduate level due to the risk of misdiagnosis, and that it distracts students’ attention away from the physical examination.^21^,^22^

A significant body of literature attests to the worldwide implementation of bedside ultrasound education at the undergraduate level in Australia,^10^,^23^–^25^ Austria,^17^ China,^26^ Germany,^11^,^14^,^27^ France,^28^ the United States,^7^,^12^,^18^,^29^–^39^ and the United Kingdom.^13^,^40^,^41^ Initial reports from Canada demonstrate the implementation of ultrasound into anatomy teaching at McMaster University in 2005,^15^,^16^ the development of an undergraduate curriculum for focused cardiac ultrasound at Queen’s University in 2013,^42^ and the integration of a four-year clinical problem-based bedside ultrasound program in the medical school at McGill University in 2013.^43^,^44^

The aim of this study was to provide a comprehensive view of the extent and characteristics of undergraduate bedside ultrasound teaching in medical schools across Canada. A survey of the similarities and differences between medical schools may inform the development of national guidelines for curricular standardization.

## Methods

The bilingual (French/English) survey was developed by a team of ultrasound experts including two clinicians certified in point-of-care ultrasound, a medical education specialist/clinician, a biomedical scientist, and a first-year medical student in April 2014. Invitations to complete the survey were sent by e-mail in May 2014 to target participants. The invitation contained a cover letter, an abstract describing the objectives and methodology of the study, and a link to the online survey. Follow-up reminders were sent by e-mail to non-respondents after four weeks and again after eight weeks. After 12 weeks, non-respondents were contacted by telephone and encouraged to complete the survey. Completed surveys were collected until December 2014, over a total of eight months.

The survey contained 19 questions divided into four sections: implementation and duration (eight questions), instructional format and approach (three questions), logistics of instruction (three questions), and administrators’ opinion regarding the role of bedside ultrasound education in the undergraduate medical school curriculum (five questions). Questions were clearly and simply worded using non-biased language and positive wording. The survey took on average 10 minutes to complete. The survey and study design were reviewed and approved by the McGill Faculty of Medicine Institutional Review Board (#A04-E34-14A). The survey was pilot tested and critically reviewed by a director of medical education at St. Mary’s Hospital Centre, a McGill University affiliated teaching hospital.

The sample size consisted of vice-deans of undergraduate medical education at accredited Canadian medical schools. The vice-deans were identified by searching the official website of each medical school.

Data were collected via online completion of the survey. The survey was distributed by SurveyMonkey (Palo Alto, California, USA). Biases relating to self-completion surveys were minimized by ensuring targeted undergraduate vice-deans had similar administrative positions and responsibilities, and similar access to a computer. Survey responses were analyzed and reported as percentages in tabular format.

The survey and study design were reviewed and approved by the McGill Faculty of Medicine Institutional Review Board (#A04-E34-14A). The survey was pilot tested and critically reviewed by a director of medical education at St. Mary’s Hospital Centre, a McGill University affiliated teaching hospital.

## Results

There are 17 LCME-accredited medical schools in Canada. The schools offer four-year medical programs except for two schools that offer three-year programs.^45^ Thirteen schools responded to the survey resulting in a 76% response rate.

### Implementation and duration

Close to 50% of the responding medical schools had implemented bedside ultrasound education in their undergraduate curriculum. Implementation was initiated primarily within the past two years between 2013–2015 (67% of schools) and occurred most often in the first two or all years of medical school (Table 1). The duration of bedside ultrasound teaching varied according to the year of medical school. A high proportion of the medical schools (67%) taught bedside ultrasound to Y3 and Y4 medical students as part of clerkship rotations, most commonly for rotations in emergency medicine but also for rotations in internal medicine (17%), intensive care (17%), and anaesthesia (17%).

### Instructional format and approach

All of the medical schools with bedside ultrasound education reported using a practical instructional format, in some cases alone or in combination with a theoretical format (Table 2). The practical format includes hands-on teaching whereby the students operate the ultrasound probe in the presence of an instructor while scanning live models or ultrasound simulators. For the instructional approach, almost all schools used a clinical problem based approach (83%), either alone or with other approaches such as procedure, anatomy, and physiology based approaches. The most common resource materials for teaching bedside ultrasound were online text and video material in combination with printed or electronic textbooks.

### Logistics

The instructors for bedside ultrasound teaching were predominantly non-radiologist physicians with recognised expertise in bedside ultrasound (Table 3). The ratio of instructors to students was most commonly 1:4 for ultrasound instruction as reported by 67% of the schools. Teaching took place in different locations, including an anatomy laboratory, a medical simulation centre, a classroom, a hospital, or a combination of these locations (Table 3).

### Administrators’ opinion

All responding vice-deans (or faculty members familiar with bedside ultrasound education) indicated that bedside ultrasound is a useful adjunct to the physical examination and that ultrasound-guided procedures improve patient safety (Table 4). The majority of vice-deans (77%) agreed that bedside ultrasound education should be part of the medical curriculum. This consensus was upheld in 57% of schools that did not teach bedside ultrasound education. Most vice-deans felt that the greatest obstacle to integrating bedside ultrasound in the medical school curriculum was the lack of ultrasound machines and infrastructure (77%).

## Discussion

The data demonstrate that approximately 50% of the 13 schools responding to a national survey of accredited medical schools in Canada had implemented undergraduate bedside ultrasound education. In the responding medical schools, bedside ultrasound teaching predominantly: 1) is implemented in all years of medical school with a duration of 1–5 hours/year, 2) is taught using a practical format and a clinical problem-based approach with a combination of textbooks and online resource materials, and 3) is taught in an anatomy laboratory or medical simulation site by non-radiologist physicians with experience in bedside ultrasound in a 1:4 instructor to student ratio. The general opinion of administrators was that ultrasound education should be integrated into the medical school curriculum.

The implementation of bedside ultrasound education in Canada is consistent with a worldwide trend for integrating undergraduate bedside ultrasound education in medical school curriculum. An initial review of the literature attests to the international implementation of undergraduate ultrasound education in universities.^7^,^10^–^18^,^23^–^44^ Bedside ultrasound education in Canadian medical schools occurs across all years of medical education, in agreement with the implementation of ultrasound education in other countries.^23^,^30^

A combination of instructional approaches observed in Canadian medical schools correlates well with medical schools in other countries. An anatomy- and physiology-based approach is used in France,^28^ while a clinical problem- and anatomy-based approach is used in Germany^27^ and the United States.^36^,^46^,^47^

The majority of vice-deans of responding Canadian medical schools stated that bedside ultrasound should be integrated into their medical school curriculum, and listed the lack of ultrasound machines and infrastructure as the most common barrier to implementation. Both of these findings are in agreement with a recent national survey of ultrasound education in medical schools in the United States.^30^

One limitation of this study is that comparisons between the responding group and the non-responding group were not amenable to statistical analyses due to the small sample size of each group. A second limitation is the possibility of a sample bias. Vice-deans of medical schools with bedside ultrasound teaching might be more likely to respond to the survey than vice-deans of medical schools without bedside ultrasound teaching.

### Conclusions

The results provide a portrait of undergraduate bedside ultrasound education in Canada for the first time. The study helps to place the implementation of Canadian teaching within the world landscape of undergraduate bedside ultrasound education.

## Figures and Tables

**Table 1:** Implementation and duration of bedside ultrasound education in Canada as reported by vice-deans of medical education at accredited medical schools, 2014

	Number of schools teaching bedside ultrasound in year of medical school*			
Implementation	All years	Y1+Y2	Y1+Y2+Y3	Y2+Y3+Y4
	2 (33)	2 (33)	1 (17)	1 (17)
**Duration of teaching/yr**	Y1	Y2	Y3	Y4
0 hrs	1 (17)	0	2 (33)	4 (67)
1–5 hrs	3 (50)	4 (67)	3 (50)	2 (33)
6–10 hrs	1 (17)	1 (17)	1 (17)	-
11–15 hrs	1 (17)	1 (17)	-	-

* Parentheses denote % of 6 responding schools

Y=year

**Table 2:** Instructional format, approach, and resources of bedside ultrasound education in medical schools in Canada as reported by vice-deans of medical education at accredited medical schools, 2014

Instruction	Number of schools^1^
**Format**	
Practical alone	2 (33)

Practical + theoretical	4 (67)

**Approach**	
Clinical problem based alone	1 (17)

Anatomy based alone	1 (17)

Clinical problem ± anatomy ± physiology ± procedure based	4 (67)

**Resources**	

Free online text/video alone	2 (33)

Printed or electronic textbook alone	1 (17)

Free online text/video + printed or electronic textbooks	3 (50)

1 Parentheses denote % of 6 responding schools

**Table 3:** Logistics of bedside ultrasound education in medical schools in Canada as reported by vice-deans of medical education at accredited medical schools, 2014

Logistics of teaching	Number of schools*
**Site**	

Anatomy laboratory alone	1 (17)

Medical simulation center alone	1 (17)

Combination of above + classroom + hospital	4 (67)

**Instructors**	
Non-radiologist physicians with ultrasound experience	3 (50)

Non-radiologist physicians with ultrasound experience + radiologists	2 (33)

Not specified	1 (17)

**Instructor to student ratio**	
1:4	4 (67)

1:4 to 1:12	2 (33)

* Parentheses denote % of 6 responding schools

**Table 4:** Administrators’ opinion of bedside ultrasound education at accredited medical schools, 2014

Administrators’ opinion	Number of schools in agreement*
Bedside ultrasound is a useful adjunct to the physical examination	13 (100)
Ultrasound-guided procedures improve patient safety	13 (100)
Bedside ultrasound could negatively impact patient safety	2 (15)
Bedside ultrasound education should be part of the medical curriculum	10 (77)
**Barriers for integrating bedside ultrasound in their medical curriculum:**	
Lack of ultrasound machines and infrastructure	10 (77)
Inadequate time in the curriculum	8 (62)
Lack of qualified instructors	5 (38)
Lack of faculty support	4 (31)
Inadequate evidence for the usefulness of bedside ultrasound	4 (31)

* Parentheses denote % of 13 responding schools
